# Effectiveness of home-based, non-exercise interventions for dementia: A systematic review

**DOI:** 10.3389/fnagi.2022.846271

**Published:** 2022-08-11

**Authors:** Davynn Gim Hoon Tan, Belinda Melody Bernadette Boo, Cheyenne Shuen Chong, Megan Michelle Ling-Li Tan, Boon-Seng Wong

**Affiliations:** ^1^Health and Social Sciences, Singapore Institute of Technology, Singapore, Singapore; ^2^Department of Physiology, National University of Singapore, Singapore, Singapore

**Keywords:** dementia, caregiver, cognition, home-based, behavioral symptoms, functional status, mood, QoL home-based intervention for dementia

## Abstract

**Introduction:**

Dementia is a neurodegenerative condition characterized by cognitive decline and increased functional dependency. With most persons living with dementia (PLWDs) residing at home, home-based interventions provide a convenient and individualized alternative for person-centered care. Most of the evidence focused on specific interventions or exercise-based activities; there remains a gap in understanding the impacts of a broader range of non-exercise interventions on PLWDs and their caregivers. This review aimed to understand the impacts of home-based, non-exercise interventions on the behavioral, functional, cognitive, and mood outcomes of PLWDs, and their caregiver's quality of life (QoL), burden and mood.

**Methods:**

Search for studies published up to June 2020 was conducted on CINAHL, PsycArticles, PubMed, SAGE Journals, Science Direct, and Web of Science. A search was also done manually based on the bibliographies of selected articles. The inclusion criteria for the systematic review were: (i) participants with a medical diagnosis of dementia, (ii) participants who resided at own home, (iii) intervention in the home setting, (iv) investigate interventions other than physical exercise, (v) randomized controlled trials (RCTs) or quasi-experimental studies, and (vi) full-text study published in English and in a peer-reviewed journal.

**Results and discussion:**

Eighteen studies consisting of 14 RCTs and 4 quasi-experimental studies were included. Interventions included were occupational therapy, cognitive rehabilitation, tailored activity program, cognitive stimulation therapy, personalized reminiscence, music therapy, reality orientation, biobehavioral and multicomponent interventions. Results were mixed, but important intervention features were highlighted. Personalized activities for PLWDs that are aligned to their interest and ability appeared to contribute to intervention effectiveness especially in reducing behavioral symptoms and improving functional status. Involvement of caregivers in interventions is another feature of effective interventions for both the PLWDs and the caregivers' QoL, provided it is not deemed demanding or challenging to the caregivers. The inclusion of caregiver's education was effective in reducing caregivers' burden, particularly when the interventions improved the PLWD's functional status.

## Introduction

Dementia is a neurodegenerative condition marked by cognitive decline that interferes with activities of daily living (World Health Organisation, 2012) as well as psychological and behavioral symptoms such as agitation and depression (Inel Manav and Simsek, 2019). Interventions for dementia largely aimed at alleviating behavioral symptoms, functional status, cognitive functions and mood (Clare et al., 2010; Brodaty and Arasaratnam, 2012; Orgeta et al., 2015; Carrion et al., 2018).

The progressive decline in cognitive functions in persons living with dementia (PLWDs) leads to a decreased functional ability to live independently overtime (Prince et al., 2013). Thus, PLWDs often have caregivers providing care to them, which is integral to the PLWDS' quality of life and mood, and in delaying institutionalization (Brodaty and Donkin, 2009). The caregivers of PLWDs often experience greater levels of stress and depression in contrast to caregivers providing care for older adults with other health conditions (Rahman et al., 2019), leading to a lower self-rated health among the PLWDs' caregivers. A higher level of burden was noted in PLWD's caregivers when the caregiving tasks were more demanding and when less benefits or positive experiences were perceived by the caregivers (Sörensen and Conwell, 2011). Given that caregiving duties greatly impact the physical and mental wellbeing of PLWDs' caregivers, it is important to identify the benefits of dementia interventions not only to the PLWDs but also to the caregivers such as quality of life or QoL (Jensen et al., 2015), burden and mood (Bessey and Walaszek, 2019). Reviews on interventions to caregivers such as education on the care of PLWDs found that it alleviates caregiver's burden and depression with small to moderate effects (Parker et al., 2008; Jensen et al., 2015) and that support groups or programs relieves caregiver's burden with a small effect (Parker et al., 2008). The current review aims to examine the effects of specifically home-based interventions for PLWDs that would encompass training and education as well as carrying out the intervention at home between the clinicians' visits. It would also examine interventions that were intended for PLWDs but may have a secondary effect on the caregivers.

Non-pharmacological interventions for dementia encompass a wide range of interventions that may be targeted toward PLWDs or their caregivers (Bessey and Walaszek, 2019) such as exercise, sensory-based interventions, cognitive stimulation, and reminiscence therapy (Meyer and O'Keefe, 2020). These interventions can be conducted in group or individually but research has shown that individualized interventions was found to be more effective for PLWDs in the community setting as compared to group interventions (Scott et al., 2019).

Considering that a majority of PLWDs reside in their homes with caregivers (World Health Organisation, 2012), it is valuable to evaluate the impact of home-based, non-pharmacological interventions to inform the care of community-dwelling PLWDs (Sampath et al., 2015). Home-based interventions improved BPSD, delay cognitive decline and reduce caregiver burden, as well as facilitate better customization of interventions to the needs and natural context of dyads to enhance person-centered care (Chung, 2013; Sampath et al., 2015; Gitlin et al., 2016; de Almeida et al., 2020). Home-based interventions also provide a convenient alternative for PLWDs who face difficulties traveling to institutions for treatment or prefer engaging in a familiar environment (Orgeta et al., 2015).

Reviews of home-based interventions had been focusing on physical exercise and its benefits to dyads (Burton et al., 2015; de Almeida et al., 2020) or specific interventions or settings (Van't Leven et al., 2013; Han et al., 2016; Carrion et al., 2018), systematic reviews on the wider range of non-exercise, home-based interventions for PLWDs are limited.

This systematic review thus aims to consolidate the evidence for the effectiveness of home-based, non-exercise interventions on PLWDs' behavioral symptoms, functional status, cognition and mood, as well as the impact on their caregivers in terms of caregivers' quality of life, their burden and mood. Factors that influence effectiveness of home-based interventions would also be synthesize to inform future intervention design.

In this review, the target population (P) is PLWDs and their caregivers. The target intervention type is home-based, non-exercise interventions for dementia. N comparator factor (C) is being considered. The outcome variables (O) are PLWDs' behavioral symptoms, functional status, cognition and mood, as well as the impact on their caregivers in terms of caregivers' quality of life, their burden and mood. The study designs (S) included are randomized controlled trials and quasi-experiments.

## Methods

**Figure 1:**
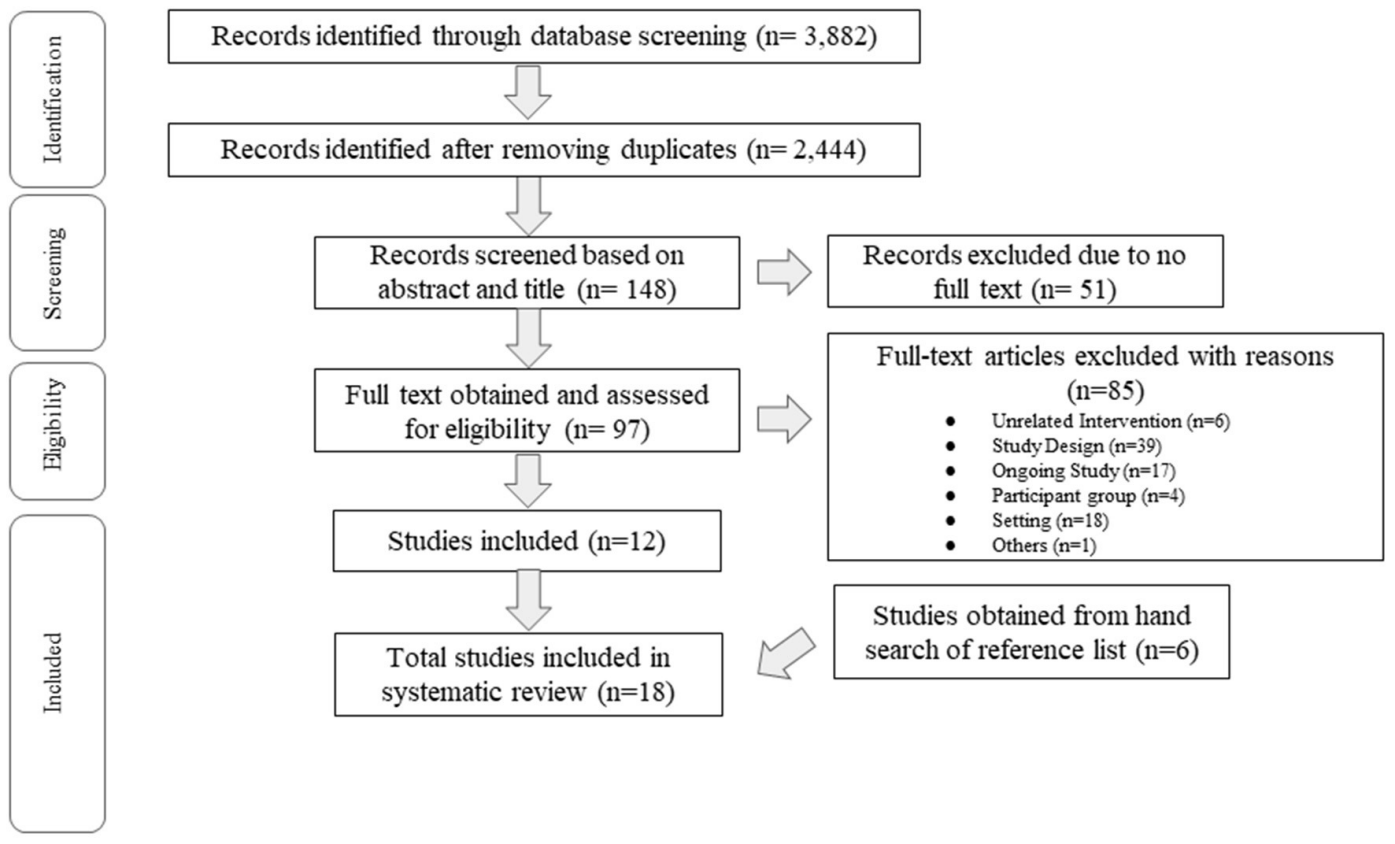
Study selection flowchart (PRISMA).

The reporting follows the Preferred Reporting Items for Systematic Reviews and Meta-Analyses (PRIMA) guideline for systematic reviews and meta-analyses (Moher et al., 2009). The flow diagram of PRISMA for this review is illustrated in Figure 1.

### Search strategy

Studies were identified and retrieved from the beginning of time up to 30 June 2021 from the following electronic databases: CINAHL, PsycArticles, PubMed, SAGE Journals, Science Direct and Web of Science. A combination of Search terms with truncation and Boolean operators (“Dementia,” “Alzheimer's Disease,” “home-based,” “home,” “home-delivered,” “individualized,” “therapeutic,” “activit^*^,” “intervention,” “engagement,” “music,” “art,” “reminiscence”) were used to identify potential articles within the databases. In addition, the reference lists of all relevant full-texts and review articles were hand-searched for eligible articles.

### Study selection

Titles and abstracts were screened according to the predefined eligibility criteria by three reviewers (BMB, CS, MML). Next, the full-text articles were screened for final inclusion in the systematic review by the same three reviewers. If no consensus was reached among the three reviewers, the plan was to discuss the case with the fourth author (DGH) until a final consensus was reached; but this option was not required.

### Eligibility criteria

The inclusion criteria for the systematic review were: (i) participants with a medical diagnosis of dementia, (ii) participants who resided at home in the community, (iii) use of individualized intervention in the home setting for participants, (iv) investigate interventions with treatment components other than physical exercise, (v) randomized controlled trials (RCTs) or quasi-experimental studies, and (vi) full-text study published in English and in a peer-reviewed journal.

Studies were excluded if (i) the intervention was conducted in group setting, (ii) the intervention was conducted in institutionalized settings, and (iii) the study focused solely on exercise-related intervention.

### Assessment of reporting quality

The quality of each included study was examined independently by two reviewers among BMB, CS, and MML using the Joanna Briggs Institute (JBI) Critical Appraisal Tool Checklist for randomized controlled trials (Joanna Briggs Institute, 2017b) and quasi-experimental studies (Joanna Briggs Institute, 2017a). The domains covered include selection bias, study design, reliability of data collection and appropriate result analysis. Next, the two reviewers for each study discuss to reach consensus over the ratings. If discrepancies occurred, the plan was to discuss with a third author until a final consensus on the ratings is reached.

### Data extraction

Relevant data on the study characteristics were independently extracted by the three reviewers (BMB, CS, MML). The characteristics extracted were experimental design, participants' demographics, intervention protocol and components, outcome measures, and findings. After independent data extraction, the accuracy of the data was verified by one other reviewer. The data was analyzed and synthesized on the qualitative directionality of the effects reported in the included studies. The effects on the PLWDs were grouped into: (i) behavioral disturbance, (ii) cognition, (iii) mood, (iv) functional status. The effect on the caregivers of PLWDs in terms of (i) quality of life, (ii) mood, and (iii) burden were also analyzed.

## Results

### Study selection

A total of 3,882 studies were identified through the screening of the different databases, with 2,444 studies remaining after duplicates were removed. After the advanced search filter (abstract and/or title) was used, 148 studies were screened, with 97 full-text studies obtained and assessed for eligibility. Eighty-five studies were excluded, with reasons such as the type of study design, ongoing studies, settings of the studies and interventions that were unrelated to the research topic. This resulted in a total of 18 studies being included. The PRISMA flow diagram for the current review is illustrated in Figure 1.

### Descriptive characteristics of the included studies

Fourteen studies were RCTs and four studies were quasi-experimental studies. Among the 18 studies included (Table 1), 1,520 PLWDs and 1,420 caregivers were investigated.^1^ Most studies included PLWD participants diagnosed with dementia generally except for 3 studies (Dooley and Hinojosa, 2004; Onder et al., 2005; Callahan et al., 2017) that investigated Alzheimer's disease (AD) and 1 study (O'Connor et al., 2019) that examined frontotemporal dementia (FTD).

**Table 1:** Study characteristics of included studies.

**Study**	**Participants**	**Regimen**	**Intervention details**	**Outcome measures**	**Findings**
Bourgeois et al. (2015) RCT	PLWD: *n* = 52 Mild to moderately severe AD (MMSE: 17.42 ± 3.77) **Trial and error learning (TE)** (*n* = 21) **Errorless learning** (*n* = 15) **Modeling with spaced retrieval** (*n* = 16)	6 weeks × 2 × 2 h	**Cognitive rehabilitation** PLWD trained on 3 IADLs based on interest and current task performance TE: PLWD tries task independently, but mistakes are corrected and cues given. EL: Giving instructions and cues before task execution to prevent mistakes made. MR: Steps of task performed in front of PLWD, and recall of steps after delay **Conducted by:** OTs	Measured at baseline, 1 week post-intervention, 4 weeks post-intervention *Primary outcomes* **Participants' performance in IADL tasks**: 3-point scale ranging from competence to deficit *Secondary outcomes* **Cognition**: MMSE **BPSD**: NPI	**IADL task scores**—Significant improvement in IADL score within group, no significant difference between groups. Participants' performances remained stable 1 month post-intervention. **Cognition, BPSD**—No significant difference within and between groups
Callahan et al. (2017) RCT	**Intervention**: Occupational therapy + Collaborative Care (TAU) (*N* = 82) **Control**: Collaborative Care (TAU) (*N* = 76)	Total duration: 24 months (18 home visits) Delivery and frequency: Cycle 1−8x 90 min session biweekly for 16 weeks Cycle 2−8x session every month for 32 weeks Cycle 3−8x session over 1 year	**Intervention components**: 1. Home modification and assessments 2. ADL and IADL training 3. Home exercise program 4. Caregiver education and training 5. Cognitive training 6. Phone calls **Conducted by**: OTs and OTAs	Measured at baseline, 6, 12, 18, 24 months *Primary outcome* **Functional status** ADCS ADL^a^ **Secondary outcomes** **Physical performance** SPPB^b^ **Sarcopenia** SPSM^c^	**Functional status** No significant difference **Physical performance and sarcopenia** Progressive functional decline, decrease in SPPB and SPSM, and decline in mean MMSE scores across time
Clare et al. (2010) RCT	PLWD: *n* = 64 Caregivers: *n* = 44 Mild AD (MMSE: 23.14 ± 3.12) Stable dose of acetylcholinesterase-inhibitor for at least 4 weeks **Intervention** Cognitive Rehabilitation (*n* = 20 PLWD, 13 caregivers)	8 weeks × 1 × 60 min	**Cognitive Rehabilitation:** 1. Teaching practical aids and strategies. 2. Introducing methods to learn new information. 3. Practice on sustaining attention and concentration. 4. Stress management techniques. 5. Carers joined the last 15 min of session to support between-session implementation **Conducted by:** 1. OTs 2. Caregivers conducted practice in between sessions	Measured at baseline, 8 weeks, 6 months post-intervention *Primary Outcome* **Goal Performance and Satisfaction:** COPM *Secondary Outcome* **Cognition:** RBMT-II, TEA. **Verbal Fluency:** TEA (Verbal fluency subtest). **Functional Status:** ILS (Health and Safety subtest), IADLS, PSMS.	**Goal Performance and Satisfaction** -Significant improvement in performance and satisfaction in CR group compared to NT and RT. Larger increase in performance noted for PLWD with caregivers involved in intervention. **PLWD anxiety and depressive symptoms**—Significant decrease within CR group, but no significant difference between all groups as decrease was seen in all groups
	**Comparison 1** Relaxation (*n* = 21 PLWD, 8 caregivers) **Comparison 2** No treatment (*n* = 23 PLWD, 7 caregivers)			**PLWD anxiety and depressive symptoms:** HADS **Quality of Life of PLWD**: QOL-AD **Caregiver Mood:** HADS **Caregiver QOL:** WHOQOL-BREF **Caregiver wellbeing:** GHQ-12 **Caregiver Burden:** RSS **Memory awareness**: MARS (Memory Functioning and Memory Performance subscales) **Brain activation:** fMRI scan during face-name association task	**Verbal fluency**—Decrease within CR and RT groups, no significant difference between groups **Caregiver QOL**—Significant improvement in WHOQOL-BREF (social relationships) within CR group. CR and RT scores significantly different to NT group, which had decline in scores **Cognition, functional status, QOL of PLWD caregiver mood, caregiver burden, caregiver wellbeing**—No significant difference within and between groups **Memory awareness**—No significant difference within CR group, but significantly better compared to NT **Brain activation**—Greater brain activation during encoding and recognition in face-name tasks in CR group than controls from RT and NT
Dooley and Hinojosa (2004) Quasi-experimental	*N* = 40 dyads No mention of group sample size. **Intervention:** Individualized occupational therapy home visits with suggestions based on Assessment of Instrumental Function (AIF) results **Control:** Mailed report with suggestions from AIF results	**Intervention group:** 2 visits (1 assessment visit to provide recommendations + 1 follow-up 30 min visit) Frequency ranged from 1 to 6 months across participants with an average of 2.33 months follow-up	**Recommendation of strategies to caregiver:** Environmental modifications Strategies for caregiver to improve ADL performance Community-based assistance **Conducted by:** OTs	Measured at baseline and follow-up **PLWD's Quality of Life (QOL)** *Positive Affect and Activity Frequency:* Affect and Activity Limitation- Alzheimer's Disease Assessment (AAL-ADD) *Self-care status*: Physical Self-Maintenance Scale **Caregiver burden** Zarit Burden Interview	**QOL** Significant improvement in aspects of positive affect, activity frequency and self-care status as compared to control **Caregiver burden** Significantly lower as compared to control
Gitlin et al. (2018) RCT	*N* = 160 dyads Diagnosis of dementia (MMSE: 16.6 ± 7.8) **Intervention** Tailored Activity Program (*n* = 76 dyads) **Control** Phone-based caregiver education, with no discussion of activity or behavioral symptoms (*n* = 84 dyads)	4 N (8 sessions, no mention on frequency or duration)	**Tailored Activity Program** (1) Assessment on PLWD and caregiver capabilities, routines, interests, environment (2) Activity prescription with goals, environment set-up, strategies for implementation and grading. (3) Caregiver education on using activities, handling distress and managing behavioral symptoms (4) Caregiver training on activity simplification and strategies for other care challenges **Conducted by:** OTs	Measured at baseline, 4, 8 months *Primary outcome* **BPSD**: NPI-C *Secondary outcomes* **Functional dependence:** CAFU **Pain during activities:** Pain Intensity Scale. **Affect of PLWD:** 6 quality of life items on a 5-point scale. **Caregiver depression:** CES-D. **Caregiver burden:** ZB (SF). **Time spent caregiving:** Number of hours providing ADL/IADL assistance, doing things for PLWD in 24-h day	**BPSD**—Significant reduction in number, and frequency × severity of BPSD symptoms within TAP group, and compared to control group **Functional dependence** No significant decrease in number of activities assisted within group, Significant difference between groups as control group had greater increase **Pain during activities**—No significant pain reduction within group, significant difference between groups as control had increase in pain **Caregiver distress**—Significant decrease in behavior-related distress, compared to control **Time spent caregiving**—No significant decline in time spent within and between groups **PLWD affect, caregiver depression, burden**—No significant difference within and between groups
Gitlin et al. (2008) RCT	*N* = 56 dyads Diagnosed wth Dementia (MMSE: 11.6 ± 8.1) **Intervention** TAP (*n* = 27 dyads) **Control** Waitlist (*n* = 29 dyads)	4 months 8 sessions−6 × 90 min home visit 2 × 15 min telephone contact with OT	**Tailored Activity Program** (1) Assessments to understand routine, abilities, interests of PLWD, communication, home environment (2) Personalized activity plan with implementation techniques (3) Caregiver education on stress reduction strategies (4) Caregiver training on activity use, adjusting activity complexity and strategies for general care problems	Measured at baseline and 4 months *Primary outcomes* **BPSD:** Scale on number and frequency of 24 behaviors, compiled from ABDS, RMBPC, previous research and report of families. **Mood:** CSDD **Activity Engagement:** Caregiver report of patient in past two weeks.	**BPSD**—Significant decrease in frequency of BPSD symptoms of TAP group compared to control. Non-significant decrease in overall number of behaviors, despite significant decrease in agitation and argumentative behaviors in TAP group. **Activity engagement**—Significant improvement in TAP group compared to control **QOL of PLWD**—Non-significant improvement in QOL in TAP group compared to control
			**Conducted by:** OTs 2. Caregivers conducted practice in between sessions	**Quality of life:** QOL-AD. **Secondary outcomes** **Caregiver Burden:** Subjective burden scale (upset with behaviors), Zarit Burden Scale, objective burden scale (time spent on PLWD matters). **Caregiver Mood:** CES-D. **Caregiver self-efficacy:** 5 item scale for confidence. **Caregiver Mastery:** 5 item scale for mastery **Caregiver skill enhancement**: TMSI	**Caregiver burden**—Significantly fewer hours spent (objective), no significant difference for subjective burden within and between groups **Mood**—No significant difference within and between groups **Caregiver mastery, self-efficacy, skill enhancement**—Significant improvement in TAP group over control **Caregiver mood**—No significant difference between groups
Gitlin et al. (2010) RCT	*n* = 209 dyads Dementia MMSE: 13.4 ± 8.1 **Intervention:** COPE biobehavioral intervention (*n* = 102) **Control**: Telephone calls with scripted questions on care challenges and strategies (*n* = 107)	4 months (10 sessions)	**Biobehavioral intervention** (1) Assessment: capabilities and deficits of patients, home environment, concerns and communication of caregivers. (2) Caregiver education of dementia and PLWD's capabilities. (3) Caregiver training: Problem solving, communication, activity engagement, task simplification and caregiver stress reduction **Conducted by:** 1. OTs 2. Advanced practice nurse	Measured at baseline, 4, 9 months *Patient outcomes* **Functional dependence:** FIM **Patient's Quality of Life (perceived by caregiver:** QOL-AD **Activity engagement:** 5-item scale. **Agitated behaviors:** ABID *Caregiver outcomes* **Caregiver wellbeing**: PCI **Caregiver confidence:** 5 investigator developed items. **Challenging problems:** Targeted measurement approach (9 months follow-up). **Caregiver appraisal of study benefits:** 11-item survey (9 months follow-up)	**Functional dependence**—Significant improvement in IADLs, non-significant improvement in ADLs between groups **Activity engagement**—Significant improvement of COPE group over control group **QOL of PLWD, agitated behaviors**—No significant difference between groups **Challenging problems**−62.7% of COPE caregivers had 1 or more caregiver-identified problems eliminated in 4 months, compared to 44.9% in control group. **Caregiver wellbeing, confidence**—Significant improvement in COPE group over control group *9*-*month* follow up: No statistical difference for any outcome measure, but COPE group caregiver perceived more benefits of intervention
Graff et al. (2006) RCT	*N* = 135 dyads **Intervention:** Occupational therapy (*N* = 68) **Control:** No Occupational therapy (*N* = 67)	**Intervention group:** 10 × 60 min (across 5 weeks)	**Intervention:** Occupational Therapy—teaching participants strategies on how to cope with cognitive decline and teaching caregivers on strategies to manage and cope with their caregiving duties. **Conducted by:** OTs	Measured at baseline, end-point (Week 6) and follow-up (Week 12) **Functional Status** Assessment of motor and process skills (AMPS), Interview of deterioration of daily activities in dementia **Caregiver's burden/sense of competence** Sense of competence questionnaire	**Functional status** Significant improvement in functional abilities as compared to control **Caregiver's burden/6 sense of competence** Significant improvement as compared to control
Holden et al. (2019) Quasi-experimental	*n* = 11 dyads Dementia MOCA: 14.4 ± 7.8 **Intervention**: Neurologic music therapy (*n* = 11)	6 weeks × 1 × 60–90 min	**Neurologic music therapy** (1) Musical sensory orientation training: sing and play-along with instrument. (2) Musical Attention Control Training: Sustained and selective attention music -based tasks (3) Associative mood and memory training: Music and memory reminiscence (4) Caregiver skills training on techniques for NMT, social and emotional interactions **Conducted by:** Music therapist	Measured at baseline, 6, 12 weeks **BPSD:** NPI-C **Quality of life:** QOL-AD **Functional disability:** DAD **Caregiver burden:** ZBI **Caregiving self-efficacy:** RSCSE	**BPSD**—Significant decrease in NPI score at 6 and 12 weeks from baseline **Caregiving self-efficacy**—No significant overall difference. Deteriorated at 6 weeks but improved at 12 weeks **QOL, Functional disability, Caregiver burden**—No significant difference
Laird et al. (2018) Quasi-experimental	*N* = 29 dyads (PLWDs: mild-moderate dementia) **Intervention**: home-based personalized reminiscence using ipad app (Inspired) (*N* = 29) **No control group**	**Intervention**: 19 weeks (5 training sessions + 12 weeks × 3)	**Intervention:** Files uploaded (photos, videos, audios) to view and access for reminiscence purposes **Conducted by**: 1. Reminiscence trainer (training) 2. Trained caregivers	Measured at baseline, midpoint (Week 13) and closure (Week 19) *Primary outcomes* **Mutuality** Mutuality scale **Secondary outcomes** **Relationship quality** Quality of Care-Patient Relationship Scale (QCPR)	**Mutuality** PLWDs: Significant increase in scores Caregivers: No significant difference **Relationship quality** PLWDs: Significant improvement Caregivers: No significant difference **Subjective wellbeing** PLWDs: Significant improvement Caregivers: Non-statistically significant decrease
				**Subjective wellbeing** World Health Organization-Five Wellbeing Index (WHO-5)	
Moniz-Cook et al. (1998) Quasi-Experiment	*n* = 30 PLWD, 20 caregivers Mild dementia MMSE: Not mentioned **Intervention** Individualized psycho-education and memory rehabilitation (*n* = 15 PLWD, *n* = 10 caregiver) **Control** Pamphlets on dementia care (*n* = 15 PLWD, *n* = 10 caregivers)	4–14 weeks (total 6–12 h)	**Psychoeducation by psychologist** Information on dementia, crisis prevention, counseling, emphasis of tapping on remaining abilities and being socially active **Individualized memory rehabilitation by caregiver** Use of psychological and practical techniques applied in daily tasks **Conducted by:** Psychologist, caregiver	*PLWD:* Measured at baseline and 18 months later (follow-up) *Caregivers:* Measured at 6 and 18 months after diagnosis **Cognition:** RBMT **Caregiver Wellbeing:** GHQ-30. **Caregiver mood:** BDI, HADS **Service usage:** Recording service usage data	**Cognition** **–** Non-significant improvement within experimental group, but significant difference compared to deterioration noted in control group. **Caregiver wellbeing and mood**—Significant improvement within and between groups **Service usage** **–** Significant difference between groups
O'Connor et al. (2019) RCT	*n* = 20 dyads Frontotemporal dementia MOCA: 12.0 ± 15.5 **Intervention** TAP (*n* = 9 dyads) **Control** Phone call (*n* = 11 dyad)	4 months (8 sessions)	**Tailored Activity Program** Assessment on abilities and interests of PLWD. 2) Personalized activity plan 3) Caregiver education about dementia, activity use, managing behaviors and adapting to declining abilities of PLWD) **Conducted by:** OTs	Measured at baseline, 4 months **BPSD:** NPI-C **Functional Status**: DAD. **Quality of life/wellbeing:** EQ-5D. **Caregiver Vigilance**: Vigilance scale	**BPSD**—Significant decrease in BPSD in TAP group than in control group, although agitation significantly worsened in TAP group **Functional status**—Significant improvement within and between groups **Quality of life, caregiver vigilance**—No significant difference within and between groups
Onder et al. (2005) RCT	*n* = 137 dyads Alzheimer's Disease MMSE: 20.2 ± 3.3 Stable dose of cholinesterase inhibitors ≥3 months **Intervention** Reality Orientation Program (*n* = 70) **Control** No treatment (*n* = 67)	25 weeks × 3 × 30 min	**Reality orientation programme** (1) Orientating to personal factors, time and location. (2) Introducing general topics. (3) Cognitive exercises on attention, memory and visuospatial ability. (4) Caregivers engaged PLWD in informal reality-based communication during day **Conducted by:** Caregivers	Measured at baseline and at 25 weeks post-intervention **Cognition:** MMSE, ADAS-Cog **Functional Status:** BI, Lawton IADL scale **BPSD:** NPI **Caregiver Mood:** HAS, HRSD **Caregiver QOL:** SF-36 **Caregiver Burden:** CBI	**Cognition**—Significant improvement in MMSE and ADAS-Cog within and between groups **BPSD, Functional status**: No significant difference within and between groups **Caregiver mood, quality of life, burden**—No significant difference within and between groups
Orgeta et al. (2015) RCT	*n* = 273 dyads Mild to moderate dementia MMSE: 21.23 ± 4.30 **Intervention** ICST (*n* = 134) **Control** TAU, which varied between centers and over time (e.g., non CST-based group activities) (*n* = 139)	25 weeks × 3 × 30 min	**Individual Cognitive Stimulation Therapy (ICST)** Each session consists of reality orientation, discussion of current events and the main ICST activity selected from two difficulty levels of cognitive demands **Conducted by**: 1. Caregivers with support from researchers (telephone, 2x home visits)	Measured at baseline, 13 and 26 weeks *Primary outcomes* **Cognition:** ADAS-Cog. **QOL of PLWD:** QOL-AD. **General health status of caregiver:** SF-12 **Secondary outcomes** **Dementia-specific QOL:** DEMQOL. **BPSD:** NPI. **Functional ability:** BADLS. **Depressive symptoms of PLWD:** GDS-15. **Anxiety and depressive symptoms of caregiver:** HADS. **Quality of caregiving relationship:** QCPR. **Health-related QOL of CG**: EQ-5D. **Resilience:** Resilience Scale (RS-14)	**Cognition, QOL, ADLs, BPSD, depressive symptoms of PLWD**—No significant difference between groups **Depressive and anxiety symptoms of caregiver, general health status of caregiver, resilience, caregiver distress**—No significant difference between groups **Quality of caregiving relationship**—Significant improvement from PLWD's perspective in ICST group over control group, but no significant difference from caregiver's perspective between groups **Health-related QOL of caregiver**—Significant improvement of ICST group over control group
Orrell et al. (2017) RCT	*n* = 273 dyads Mild to moderate dementia MMSE: 21.12 ± 4.48 **Intervention** ICST (*n* = 134) **Control** TAU, which varied between centers and over time (e.g., non-CST-based group activities) (*n* = 139)	25 weeks × 3 × 30 min	**Individual Cognitive Stimulation Therapy (ICST)** Each session consists of reality orientation, discussion of current events and the main ICST activity selected from two difficulty levels of cognitive demands **Conducted by**: Caregivers with support from researchers (telephone, 2x home visits)	Measured at baseline, 13 and 26 weeks *Primary outcomes* **Cognition:** ADAS-Cog. **QOL of PLWD:** QOL-AD. **General health of caregiver:** SF-12 **Secondary outcomes** **Dementia-specific QOL:** DEMQOL. **BPSD:** NPI. **Functional ability:** BADLS. **Depressive symptoms of PLWD:** GDS-15. **Anxiety and depressive symptoms of caregiver:** HADS. **Quality of caregiving relationship:** QCPR. **Health-related QOL of CG**: EQ-5D. **Resilience:** Resilience Scale (RS-14)	**Cognition, QOL, ADLs, BPSD, depressive symptoms of PLWD**—No significant difference between groups **Depressive and anxiety symptoms of caregiver, general health status of caregiver, resilience, carer distress**—No significant difference between groups **Quality of caregiving relationship**—Significant improvement from PLWD's perspective in ICST group over control group, but no significant difference from caregiver's perspective between groups **Health-related QOL of caregiver**—Significant improvement of ICST group over control group
Prick et al. (2016) RCT	*n* = 111 dyads Mild to moderate dementia MMSE: 21 ± 5.19 **Intervention** Multicomponent intervention (*n* = 57) **Control** Bulletins (*n* = 54)	3 months (8 sessions) 1st month: 4 weeks × 60 min 2nd and 3rd month: 2 weeks × 60 minutes	**Multi-component intervention** Physical exercise. (2) Psycho-education. (3) Communication Skills training. (4) Pleasant activities training **Conducted by:** 1. Research personnels 2. Caregivers (conduct homework activities between sessions)	Measured at baseline, 3 months (end-point), 6 months *Primary outcomes* **Mood (Depression):** CSDD, DRS-RAI-HC. **Physical health:** SF-36, SIP **Secondary outcomes** **Behavioral disturbance:** RMBPC	**Mood**—Significant increase in depressive symptoms compared to control (DRS-RAI-HC), no significant difference (CSDD). However, intervention participants expressed pleasure gained from intervention. **Behavioral disturbance**—Increase at end-point and follow-up, compared to control **Physical health**: No significant difference within and between groups
Prick et al. (2017) RCT	*n* = 111 dyads Mild to moderate dementia MMSE: 21 ± 5.19 **Intervention** Multicomponent intervention (*n* = 57) **Control** Bulletins (*n* = 54)	3 months (8 sessions) 1st month: 4 weeks × 60 min 2nd and 3rd month: 2 weeks × 60 minutes	**Multi-component intervention** (1) Physical exercise. (2) Psycho-education. (3) Communication Skills training. (4) Pleasant activities training. **Conducted by:** 1. Research personnel 2. Caregivers (conduct homework activities between sessions)	Measured at baseline, 3, 6 months **Cognition** Memory: 8WT, RBMT. Executive function: WMS-R, BADS, GIT. Attention: WMS-R (Digit Span Test Forward subtest)	**Attention**—Small significant effect in intervention group over control group **Memory and executive function**—No significant difference between groups
Thivierge et al. (2014) RCT	*n* = 17 PLWDs Mild to moderate AD MMSE: 21.56 ± 2.51 **Intervention**: Cognitive rehabilitation (*n* = 9) **Control**: Waiting list (*n* = 8)	4 weeks × 2 × 45–60 min	**Cognitive rehabilitation:** Train IADL based on dyad's needs and interests. Errorless learning: Adjusting degrees of assistance given to reduce errors Spaced retrieval: increase in delays given between correct realization of task **Conducted by:** 1. Trained research personnel 2. Caregivers (conduct practices between sessions)	Measured at baseline, 5, 9, 13 weeks *Primary outcomes* **IADL performance:** DMT **Secondary outcomes** **Cognitive status:** DRS-2 **Everyday memory function:** RBMT **Behavioral symptoms**: NPI **Functional disability:** DAD **Quality of Life:** DEMQoL **Caregiver burden:** ZBI-22	**IADL performance**—Significant improvement within intervention group and over control group, maintained until follow-up at week 13. **Cognitive status, memory, behavioral symptoms, functional disability, QOL, caregiver burden**—No significant difference within and between groups

a ADCS ADL: Alzheimer's disease cooperative study group activities of daily living scale.

b SPPB: Short physical performance battery.

c SPSM: Short portable sarcopenia measure.

The studies covered interventions that include cognitive rehabilitation (*n* = 3), tailored activity programme or TAP (*n* = 3), cognitive stimulation, (*n* = 2), occupational therapy (*n* = 2), reminiscence (*n* = 1), music therapy (*n* = 1), reality orientation (*n* = 1), biobehavioral intervention (*n* = 1), and multi-component intervention (*n* = 4).

### Reporting quality

The reporting quality of the 14 RCTs and the 4 quasi-experiments as evaluated by JBI Critical Appraisal Checklists of the relevant study designs are presented in Tables 2, 3, respectively. Among the RCTs, majority of the studies did not explicitly report the blinding of participants and the staff who deliver the interventions (Table 2). However, the outcome assessors in all studies were blinded except Gitlin et al. (2010) which was unclear. Both RCTs and quasi-experimental studies were mostly unclear in reporting the reliability of the data collection (Tables 2, 3).

**Table 2:** Quality assessment of included studies using *JBI critical appraisal checklist on randomized control trials*.

	**Bourgeois et al. (2015)**	**Callahan et al. (2017)**	**Clare et al. (2010)**	**Gitlin et al. (2008)**	**Gitlin et al. (2010)**	**Gitlin et al. (2018)**	**Graff et al. (2006)**
Randomization for assignment	Yes	Yes	Yes	Yes	Yes	Yes	Yes
Concealed allocation to treatment groups	Unclear	Yes	Unclear	Unclear	Yes	Yes	Unclear
Similar treatment groups at baseline	Yes	Yes	Yes	Yes	Yes	Unclear	Yes
Blinding of participants to treatment assignments	No	No	No	No	No	No	No
Blinding of assignments of those who deliver treatment	No	No	No	No	No	No	No
Blinding of outcome assessors	Yes	Yes	Yes	Yes	Unclear	Yes	Yes
Identical treatment of treatment groups other than intervention	Yes	Unclear	Yes	Yes	Yes	Unclear	Yes
Completion of follow-up or adequate analysis of differences in follow up	Yes	Yes	Yes	Yes	Yes	Yes	Yes
Participants were analyzed in the group they were randomized in	Yes	Yes	Yes	Yes	Yes	Yes	Yes
Outcomes measured in same way for treatment groups	Yes	Yes	Yes	Yes	Yes	Yes	Yes
Outcomes measured in a reliable way	Unclear	Yes	Yes	Yes	Unclear	Yes	Unclear
Appropriate use of statistical analysis	Yes	Yes	Yes	Yes	Yes	Yes	Yes
Appropriate trial design	Yes	Yes	Yes	Yes	Yes	Yes	Yes
**Checklist criteria**	**O'Connor et al. (** **2019** **)**	**Onder et al. (** **2005** **)**	**Orgeta et al. (** **2015** **)**	**Orrell et al. (** **2017** **)**	**Prick et al. (** **2016** **)**	**Prick et al. (** **2017** **)**	**Thivierge et al. (** **2014** **)**
Randomization for assignment	Yes	Yes	Yes	Yes	Yes	Yes	Yes
Concealed allocation to treatment groups	Yes	Unclear	Yes	Yes	Yes	Yes	Unclear
Similar treatment groups at baseline	No	Yes	Yes	Yes	Yes	Yes	Yes
Blinding of participants to treatment assignments	No	No	No	No	No	No	No
Blinding of assignments of those who deliver treatment	No	No	No	No	No	No	No
Blinding of outcome assessors	Yes	Yes	Yes	Yes	Yes	Yes	Yes
Identical treatment of treatment groups other than intervention	Yes	Yes	Yes	Yes	Yes	Yes	Yes
Completion of follow-up or adequate analysis of differences in follow up	Yes	Yes	Yes	Yes	Yes	Yes	Yes
Participants were analyzed in the group they were randomized in	Yes	Yes	Yes	Yes	Yes	Yes	Yes
Outcomes measured in same way for treatment groups	Yes	Yes	Yes	Yes	Yes	Yes	Yes
Outcomes measured in a reliable way	Unclear	Unclear	Unclear	Unclear	Yes	Yes	Unclear
Appropriate use of statistical analysis	Yes	Yes	Yes	Yes	Yes	Yes	Yes
Appropriate trial design	Yes	Yes	Yes	Yes	Yes	Yes	Yes

**Table 3:** Quality assessment of included studies using J*BI critical appraisal checklist on quasi-experimental studies*.

**Checklist criteria**	**Dooley and Hinojosa (2004)**	**Holden et al. (2019)**	**Laird et al. (2018)**	**Moniz-Cook et al. (1998)**
Is it clear in the study what is the “cause” and what is the “effect”?	Yes	Yes	Yes	Yes
Were the participants included in any comparisons similar?	Yes	Yes	Yes	Yes
Were the participants included in any comparisons receiving similar treatment/care, other than the exposure or intervention of interest?	Unclear	Yes	Yes	Yes
Was there a control group?	Yes	No	No	Yes
Were there multiple measurements of the outcome both pre and post the intervention/exposure?	Yes	Yes	Yes	Yes
Was follow up complete and if not, were differences between groups in terms of their follow up adequately described and analyzed?	Yes	Yes	Yes	Yes
Were the outcomes of participants included in any comparisons measured in the same way?	Yes	Yes	Yes	Yes
Were outcomes measured in a reliable way?	Yes	Unclear	Unclear	Unclear
Was appropriate statistical analysis used?	Yes	Yes	Yes	Yes

### Outcome on behavioral disturbance

A total of 10 RCTs (Onder et al., 2005; Gitlin et al., 2008, 2010, 2018; Clare et al., 2010; Bourgeois et al., 2015; Orgeta et al., 2015; Prick et al., 2016; Orrell et al., 2017; O'Connor et al., 2019) and 1 quasi-experimental study (Holden et al., 2019) investigated the effect of the interventions on behavioral disturbance.

The interventions included tailored activity programme (TAP), music therapy, ICST, reality orientation, cognitive rehabilitation and multicomponent interventions. Outcome measures consisted of Neuropsychiatric Inventory, Revised Memory and Behavior Problem Checklist, and Agitated Behavior in Dementia Scale.

Studies using TAP reported significant reduction of behavioral disturbance when compared to the control groups (Gitlin et al., 2008, 2018; O'Connor et al., 2019) and relative to baseline (Gitlin et al., 2018). Holden et al. (2019) found music therapy significantly reduced the behavioral disturbance in PLWD relative to baseline. Specific to agitation, two studies counterintuitively did not observe reduced agitation despite a reduction in behavioral disturbance with biobehavioral intervention (Gitlin et al., 2010) and TAP (O'Connor et al., 2019). A caveat was that Gitlin et al. (2008) did find a significant decrease in the informant-reported agitation while using the same TAP intervention as Gitlin et al. (2010). The other six studies reported no significant effect (Onder et al., 2005; Clare et al., 2010; Bourgeois et al., 2015; Orgeta et al., 2015; Prick et al., 2016; Orrell et al., 2017).

Notably, the common features among effective interventions observed among these studies included the use of activities tailored to the interests and capabilities of the PLWD, and assessments on context and needs of the dyads (Gitlin et al., 2008, 2018; Holden et al., 2019; O'Connor et al., 2019). Psychoeducation and skills training for caregivers in communication and task simplification were also prominent features of effective interventions.

There is a trend where studies that did not primarily target behavioral disturbance yielded no significant impact despite improvements in cognition or functional abilities. In contrast, interventions designed to address behavioral symptoms as the primary outcome showed significant improvements (Gitlin et al., 2008, 2018; Holden et al., 2019; O'Connor et al., 2019).

In essence, mixed findings were observed on the impact of home-based, non-exercise interventions on behavioral disturbance in PLWD. Studies that primarily targeted behavioral disturbance exhibited better effect. Effective interventions incorporated dyadic needs assessment and tailored interventions based on the interests and abilities of the PLWDs.

### Outcome on functional status

Functional status was investigated in 11 RCTs (Onder et al., 2005; Graff et al., 2006; Clare et al., 2010; Gitlin et al., 2010, 2018; Thivierge et al., 2014; Bourgeois et al., 2015; Orgeta et al., 2015; Callahan et al., 2017; Orrell et al., 2017; O'Connor et al., 2019) and two were quasi-experimental studies (Dooley and Hinojosa, 2004; Holden et al., 2019).

Outcome measures used in the studies include: Physical Self-Maintenance Scale (PSMS), Caregiver Assessment of Function and Upset Scale (CAFU), Bristol Activities of Daily Living Scale (BADLS), Alzheimer's Disease Cooperative Study Group Activities of Daily Living Scale (ADCS ADL), Functional Independence Measure (FIM), Direct Measure of Training (DMT), Disability Assessment for Dementia (DAD), Independent Living Scale (ILS), Instrumental Activity of Daily Living Scale (IADLS), Barthel Index (BI), Assessment of Motor and Process Skills (AMPS), Interview of Deterioration of Daily Activities in Dementia (IDDD) and a 3-point scale on competence, questionable and ineffective steps and deficits.

The interventions included in these studies were TAP (Gitlin et al., 2018; O'Connor et al., 2019), ICST (Orgeta et al., 2015; Orrell et al., 2017), occupational therapy (Dooley and Hinojosa, 2004; Graff et al., 2006; Callahan et al., 2017), biobehavioral intervention (Gitlin et al., 2010), cognitive rehabilitation (Clare et al., 2010; Thivierge et al., 2014; Bourgeois et al., 2015), music therapy (Holden et al., 2019), and reality orientation program (Onder et al., 2005).

Six studies reported significant improvement in functional abilities in intervention groups relative to the control groups (Dooley and Hinojosa, 2004; Graff et al., 2006; Gitlin et al., 2010, 2018; Bourgeois et al., 2015; O'Connor et al., 2019). Specifically, three of them reported improvement in functional dependence in IADLs (Gitlin et al., 2010; Bourgeois et al., 2015; O'Connor et al., 2019). Notably, all six studies conducted pre-intervention assessments to identify PLWDs' current abilities, used activities aligned to PLWDs' interests in the interventions, and teaching specific skills and strategies to both PLWDs and their caregivers to perform the functional activities.

One study reported mixed results of the impact on functional status in the cognitive rehabilitation group relative to the control group as measured in DAD (Thivierge et al., 2014) yet a significant difference in IADL performance was found measured by DMT. One caveat is that the intervention focused on training a particular IADL that was chosen by the patient and caregiver.

Six studies reported no statistically significant difference in functional status in intervention groups relative to control groups (Onder et al., 2005; Clare et al., 2010; Orgeta et al., 2015; Callahan et al., 2017; Orrell et al., 2017) and relative to baseline (Holden et al., 2019). Three studies had interventions conducted by caregivers (Onder et al., 2005; Orgeta et al., 2015; Orrell et al., 2017) and the remaining three by therapists (Clare et al., 2010; Callahan et al., 2017; Holden et al., 2019). The interventions carried out by caregivers were all conducted over 25 weeks, and caregivers were required to learn skills and techniques to facilitate engagement of activities with PLWDs. Two of them (Orgeta et al., 2015; Orrell et al., 2017) had therapists providing support to the caregivers through telephone calls and provided two monitoring visits throughout the intervention periods. The remaining study (Onder et al., 2005) had no support provided to the caregivers.

Overall, there is mixed evidence of home-based, non-exercise interventions impacting on the functional status of PLWDs but interventions that showed significant benefit were individualized to PLWDs based on their interests and current abilities, and conducted by the therapists.

### Outcome on cognition

Seven RCTs (Onder et al., 2005; Clare et al., 2010; Thivierge et al., 2014; Bourgeois et al., 2015; Orgeta et al., 2015; Orrell et al., 2017; Prick et al., 2017) and one quasi-experimental study (Moniz-Cook et al., 1998) examined the effect of the interventions on cognition.

The interventions included individualized cognitive stimulation therapy (ICST), cognitive rehabilitation, reality orientation, memory rehabilitation with caregiver psychoeducation, and a multicomponent intervention. Outcome measures used across studies were the Rivermead Behavioral Memory Test, Alzheimer's Disease Assessment Scale—Cognition, MMSE, Weschler Memory Scale-Revised, Behavioral Assessment of the Dysexecutive Syndrome, Dementia Rating Scale-2, Test of Everyday Attention, 8-Words Test and Memory Awareness Rating Scale.

Significant benefit to cognition were reported in two studies (Moniz-Cook et al., 1998; Onder et al., 2005). In Moniz-Cook et al. (1998), participants who underwent memory rehabilitation with psychoeducation performed better on memory outcomes compared to the control group. Onder et al. (2005) also reported a significant improvement in general cognition for reality orientation intervention participants within and across groups. Mixed results were found in two studies (Clare et al., 2010; Prick et al., 2017). Prick et al. (2017) reported a small significant effect on attention but no effect for executive function and memory in the multicomponent intervention group, compared to control participants. Clare et al. (2010) noted greater brain activation for encoding and retrieval in face-name tasks during fMRI scanning and higher memory self-ratings for participants who underwent cognitive rehabilitation, but no improvements were reflected in the objective assessments for memory and attention.

Common features of these interventions that yielded either significant or mixed results include reinforcement of strategies between sessions and involvement of caregivers in delivering interventions and their interventions lasting no <8 weeks (Moniz-Cook et al., 1998; Onder et al., 2005; Clare et al., 2010). All eight studies had interventions with duration of 8 weeks or more except Bourgeois et al. (2015) and Thivierge et al. (2014).

No significant effect on cognition was observed studies with home-based ICST interventions (Orgeta et al., 2015; Orrell et al., 2017). Cognitive rehabilitation studies that focused on specific learning techniques also did not appear to improve general cognition as a secondary outcome, despite improvements in primary outcomes of functional ability (Thivierge et al., 2014; Bourgeois et al., 2015).

In summary, there is mixed evidence of home-based, non-exercise interventions impacting on cognition in PLWDs. Studies with longer durations and incorporated reinforcement of strategies and involvement of caregivers in interventions appear beneficial, while ICST and cognitive rehabilitation interventions showed no effect on cognition.

### Outcome on mood

Six RCTs investigated the impact on mood by TAP (Gitlin et al., 2008, 2018), ICST (Orgeta et al., 2015; Orrell et al., 2017), cognitive rehabilitation (Clare et al., 2010) and multi-component intervention (Prick et al., 2016). Outcome measures included Geriatric Depression Scale-15, Cornell Scale for Depression in Dementia, Depression Rating Scale of the Resident Assessment Instrument Home Care, and Hospital Anxiety and Depression Scale.

There was no significant effect on mood relative to comparison groups reported although two studies found significant within-group improvements (Clare et al., 2010; Gitlin et al., 2018). Both TAP and control groups demonstrated improved caregiver-rated mood of PLWDs in Gitlin et al. (2018) whereas Clare et al. (2010) reported reduced anxiety across all 3 groups of cognitive rehabilitation, relaxation therapy and no treatment. The latter finding was attributed to the gradual habituation of participants to the assessment process. Notably, Prick et al. (2016) found worse depression scores in the intervention group, who found intervention tasks too challenging.

It is observed that participants in all studies generally did not have high depression or anxiety scores at baseline. There was also no outcome measures for positive mood used in the studies although pleasure was expressed by some participants in intervention group (Prick et al., 2016).

Limited evidence of the impact of home-based non-exercise interventions on mood in PLWDs was found albeit mood appeared to be negatively affected by challenging tasks. Outcome measures on positive emotions were also lacking across all studies.

### Outcome on caregivers

Apart from examining the effectiveness of home-based, non-exercise interventions on PLWD, three caregiver outcomes were also reviewed, namely, caregiver's QoL, burden and mood.

#### Caregivers' QoL

Caregivers' QoL were examined in five RCTs (Onder et al., 2005; Clare et al., 2010; Gitlin et al., 2010; Orgeta et al., 2015; Orrell et al., 2017), and two quasi-experimental studies (Moniz-Cook et al., 1998; Laird et al., 2018). The outcome measures used include the 12-item and the 36-item Short-Form Health Survey (SF-12, SF-36, respectively), European Quality of Life−5 Dimensions (EQ-5D), 12-item and 30-item General Health Questionnaire (GHQ-12, GHQ-30, respectively), Perceived Change Index (PCI), World Health Organization—Five Wellbeing Index (WHO-5) and the World Health Organization of Life Assessment short version (WHOQOL-BREF).

Two studies found significantly higher QoL in caregivers from the intervention groups relative to the control groups after receiving the COPE biobehavioral intervention (Gitlin et al., 2010) and a brief multi-component intervention provided to dyads before they were referred to the EMI team support services (Moniz-Cook et al., 1998). The interventions lasted from 1–4 months and conducted once a week. Both studies involved the caregivers in the interventions and Gitlin et al. (2010) had the caregivers carried out activities with the PLWD.

Three studies reported mixed findings when between-group analyses were performed: Clare et al. (2010) observed significant improvements in caregivers' QoL only in the social relationships domain of WHOQOL-BREF after the cognitive rehabilitation whereas the other two studies found significant improvements in EQ-5D but not in SF-12 among the caregivers after ICST (Orgeta et al., 2015; Orrell et al., 2017). The interventions lasted between 8 and 25 weeks and conducted once weekly by a therapist (Clare et al., 2010) or thrice weekly by a research staff (Orgeta et al., 2015; Orrell et al., 2017).

The remaining two studies observed no significant benefits in either within-group analysis (Laird et al., 2018) or between-group analysis (Onder et al., 2005). In these two studies, the interventions were personalized reminiscence using an iPad (Laird et al., 2018) and reality orientation program (Onder et al., 2005), which lasted between 19 and 25 weeks and were conducted thrice weekly with the involvement of caregivers. Counterintuitively, Laird et al. (2018) observed a decrease in caregivers' QoL albeit not statistically significant after the reminiscence intervention using iPad and notably the mean age of these caregivers was 67 years old. The caregivers were tasked to set-up and operate the iPad (Laird et al., 2018). It is unknown if the decreased QoL at post-intervention is related to the use of iPad as the study did not have a control group.

In summary, the evidence of home-based, non-exercise intervention improving the caregivers' QoL is inconclusive. Studies that showed effectiveness had actively involved caregivers in the interventions that were not overly intensive or causing inconvenience to the caregivers.

#### Caregivers' burden

Among the included studies, six RCTs (Onder et al., 2005; Graff et al., 2006; Gitlin et al., 2008, 2018; Clare et al., 2010; Thivierge et al., 2014) and two quasi-experimental studies (Dooley and Hinojosa, 2004; Holden et al., 2019) examined the caregivers' outcomes. Outcome measures used include the Zarit Burden Interview, Relatives Stress Scale, Caregiver Burden Inventory, Sense of Competence Questionnaire, a rating scale for upset with behavior; and using the caregiver's estimate of time spent on care duties as a measure of burden.

Two studies observed a significant decrease in caregiver burden between the control and intervention group after receiving individualized occupational therapy (Dooley and Hinojosa, 2004; Graff et al., 2006). Caregiver education was included in the interventions to teach caregivers care strategies and coping strategies. Both studies also observed positive impact on the functional abilities of the PLWD.

Gitlin et al. (2008) found mixed results where there was a significant reduction in objective caregiver burden (time spent on care duties) in the caregivers of the TAP group relative to the control group but showed no significant effect on subjective caregiver burden. The intervention focused on engaging PLWD in activities and training caregivers on how to carry out those activities (Gitlin et al., 2008).

The remaining five studies reported no significant effect of the interventions on caregivers' burden either in within-group (Holden et al., 2019) or between-group analyses (Onder et al., 2005; Clare et al., 2010; Thivierge et al., 2014; Gitlin et al., 2018). The interventions include TAP (Gitlin et al., 2018), neurologic music therapy (Holden et al., 2019), cognitive rehabilitation (Clare et al., 2010; Thivierge et al., 2014), and reality orientation program (Onder et al., 2005). All the interventions primarily focused on engaging PLWD in activities or training caregivers to conduct activities with the PLWD; and they did not observe any significant effect on the functional abilities of PLWD except a low functional dependence in the intervention group relative to the control group at post-intervention in the study by Gitlin et al. (2018).

Overall, most of the studies that investigated caregivers' burden reported no significant effect. Interventions that were effective had included caregiver education and concurrently yielded a positive impact on the functional abilities of the PLWDs.

#### Caregivers' mood

Caregivers' mood was measured in six RCTs (Onder et al., 2005; Gitlin et al., 2008, 2018; Clare et al., 2010; Orgeta et al., 2015; Orrell et al., 2017) and one quasi-experimental study (Moniz-Cook et al., 1998). Outcome measures used include the Centers for Epidemiologic Study Depression Scale (CES-D), Hospital Anxiety and Depression Scale (HADS), Hamilton Rating Scale for Depression (HDRS), Hamilton Anxiety Scale (HAM-A), and Beck Depression Inventory (BDI).

Only Moniz-Cook et al. (1998) observed a deterioration of mood among caregivers in the control group at 18th month post-referral relative to 6th month post-referral on BDI, GHD, HAD Anxiety and HAD Depression whereas caregivers in the intervention group remained fairly stable. A caveat is that the authors only obtained 3 caregivers' data at baseline (referral), hence a pre-intervention baseline was impossible. The two time-points of measurement for caregivers were 6-month post-referral (~3 months after intervention) and 18th month post-referral. The intervention was a multi-component intervention for the dyad prior to receiving support services from the EMI team and lasted from 4 to 14 weeks (Moniz-Cook et al., 1998).

The interventions of the remaining six studies that observed no significant benefits on the caregivers' mood were TAP (Gitlin et al., 2008, 2018), ICST (Orgeta et al., 2015; Orrell et al., 2017), cognitive rehabilitation (Clare et al., 2010), and a reality orientation program (Onder et al., 2005) and the intervention lasted from 8 to 25 weeks.

Overall, majority of the studies reported no significant benefits on caregivers' mood. A significant impact was observed when the follow-up was relatively long (18 months) albeit the direction of change was counterintuitive.

## Discussion

With a growing need to provide interventions for home-dwelling PLWDs, this review is the first of its kind to investigate the impact of home-based, non-exercise interventions on the behavioral disturbance, functional status, cognition and mood of PLWDs as well as caregivers' QoL, burden and mood.

Tailored activities for PLWDs that are aligned to their interests and abilities appeared to contribute to intervention effectiveness especially in reducing behavioral disturbance and improving functional status. Involvement of caregivers in interventions is another feature of effective interventions for both the PLWDs and the caregivers' QoL and burden, particularly when the interventions are improving the PLWDs' functional status and provided the involvement is not deemed demanding or challenging to the caregivers. Longer duration of intervention and measurement are also critical in observing effectiveness.

### Tailored activities aligned to PLWDs' interests and abilities

A reduced number and frequency of behavioral symptoms were reported in studies where the interventions were tailored toward the interests and abilities of the PLWDs (Gitlin et al., 2008, 2018; O'Connor et al., 2019). This finding corroborated with the recommendation from Brodaty and Arasaratnam (2012) for successful behavioral disturbance interventions to be tailored toward the PLWDs' needs. It has been suggested that activity engagement aligned to interests can provide meaning and support the self-identity of PLWDs, which is often an unmet need in PLWDs and resultingly manifested as BPSD (Scales et al., 2018). These activities also serve as an outlet of constructive self-expression that compensate for use of distressing behaviors to express frustration (Scales et al., 2018). Additionally, adapting difficulty of tasks to capabilities of PLWDs helps to decrease the overload of sensory and information processing required by PLWDs with reduced stress thresholds (Gitlin et al., 2018). Hence, this could minimize challenging behaviors triggered as responses to physiological stress or frustration.

Also, studies that reported significant improvement in functional abilities in the intervention groups as compared to the control groups was observed to have interventions that were individualized to PLWDs with activities chosen based on their interests and matched to their current abilities (Dooley and Hinojosa, 2004; Graff et al., 2006; Gitlin et al., 2010, 2018; Bourgeois et al., 2015; O'Connor et al., 2019). Such activities were more familiar to the PLWDs, thus allowing them to be purposefully engaged in the activities and relate better. In addition, activities chosen were modified to PLWDs' current abilities and with specific skills and strategies individualized to PLWDs, allowing PLWDs to feel more competent when engaging in the activities. The finding corroborates with a systematic review that similarly reported improved functional decline after receiving interventions planned with considerations of PLWDs' interests and abilities (Bennett et al., 2019).

### Involvement of caregivers in interventions

Interventions that included caregivers' psychoeducation or skill training reduced the number and frequency of behavioral symptoms (Gitlin et al., 2008, 2018; O'Connor et al., 2019), corroborating with the review of Brodaty and Arasaratnam (2012) and the finding of Van't Leven et al. (2013). Psychoeducation equips caregivers with the knowledge to identify triggers, symptoms and strategies to alleviate the behavioral disturbance. Hulme et al. (2010) affirmed that caregivers need to be well-informed of the behavioral symptoms and its causes besides learning strategies in order to better manage the behavioral disturbance daily. Skills training in task simplification and communication also empower caregivers to support the PLWDs' abilities and preferences in activity engagement and social interaction (Scales et al., 2018). This potentially helps to circumvent the triggers and unmet needs faced by PLWDs that result in behavioral disturbance.

Effects on cognition were also observed from interventions that involved caregivers to deliver parts of the interventions (Moniz-Cook et al., 1998; Onder et al., 2005; Clare et al., 2010), concurring with the review on dyadic interventions by Grandmaison and Simard (2003). As caregivers were familiar with the characteristics and contexts of the PLWDs, it was integral to involve them when tailoring interventions for the PLWDs. For example, caregivers in the intervention group in Onder et al. (2005) incorporated personally-relevant elements during reality orientation which aided memory and promoted cognitive gains among intervention PLWDs. Being in close contact with the PLWDs, caregivers were also the most suited to conduct regular practice of content or strategies with PLWDs at home. This constant rehearsal potentially improved short-term memory retention which might benefit orientation and recollection of new information in PLWDs (Moore et al., 2001).

Besides having positive effect on behavioral disturbance and cognition of PLWDs, actively involving caregivers is a common feature of interventions that impact on caregivers' QoL. Orgeta et al. (2015) attributed the phenomenon to the increased number of enjoyable events and experience together with the PLWDs from the involvement in the intervention and thus contributed to an improved caregivers' QoL. This explanation was supported by Vellone et al. (2012) who stated that improved QoL were seen amongst caregivers who had experienced fulfillment and gains from the caregiving.

Interventions that involve the caregivers actively also impact on caregivers' level of burden. Marim et al. (2013) had previously noted that interventions which provided education and support to caregivers were more effective in decreasing burden levels. When caregivers are equipped with knowledge on care and coping strategies, they would likely be able to cope better with care duties (Chiu et al., 2013). The increased competency helped to lower the levels of burden experienced (Palacio et al., 2018). This is corroborated with caregivers' report on the importance of caregiver education in helping them provide better care for PLWDs (Muangpaisan et al., 2010).

### Caveats of involving caregivers in interventions

Involvement of caregivers are not always beneficial. Note worthily, interventions that had significant positive effects on caregivers' burden were observe to have significant effects on the functional abilities of PLWDs too. This relationship between the two factors is unsurprising, considering that the functional ability of PLWDs is known to have an impact on carer burden levels (Chiao et al., 2015). When PLWDs have better functional abilities, they require less assistance and thus decrease the caregivers' workload. The decreased workload helped to lower burden levels as they were observed to have a linear relationship (Pinquart and Sörensen, 2003; Lethin et al., 2018).

However, Gitlin et al. (2018) found contrary result where improvements were observed in the PLWDs' functional abilities but not in caregivers' burden. One possible explanation is that the range of customized TAP activities might have led to improvements in the PLWDs' functional abilities but resulted in increased caregivers' workload that did not alleviate caregivers' burden (Pinquart and Sörensen, 2003; Lethin et al., 2018). Similar phenomenon is observed where interventions that did not have any significant effect on caregiver QoL or wellbeing have arguably also involved caregivers (Onder et al., 2005; Laird et al., 2018); the interventions were deemed as demanding or inconvenient for caregivers. In terms of intervention intensity, sessions were carried out thrice weekly, in comparison to approximately once weekly for interventions that showed significant improvements. The higher intensity might have meant increased work for caregivers, which possibly explains why no significant improvement in their QoL was seen. This is supported by Farina et al. (2017) who highlighted that some studies had found factors such as increased workload and time spent on caregiving duties had resulted in decreased QoL. In addition to the increased intensity, Laird et al. (2018) had also used a technological device in the intervention: caregivers with a mean age of 67 were tasked to handle and set-up the iPad. In comparison to younger counterparts, it has been found that older adults tend to perceive the use of technology to be more challenging (Hauk et al., 2018). Hence, the challenge of using an iPad could have caused distress to the aged caregivers which did not benefit their QoL. However, it is unknown if other factors might have contributed to the results in Laird et al. (2018) as there was no control group in the study.

In addition, interventions that mainly focused on the PLWDs' activity engagement or in training caregivers to engage PLWDs in activities yielded no significant effect on caregivers' burden. Such caregivers training differs qualitatively from the caregivers education on caring strategies and coping strategies which was deemed as helpful as mentioned previously. In addition, these studies that focused on activity engagement had also mostly seen no significant improvement in the PLWD's functional abilities which in turn impacted caregivers' burden as mentioned earlier (Chiao et al., 2015).

### Duration of intervention and measurement matters

It was observed that most of the included studies that measured cognition had interventions lasting no less than 8 weeks especially those that showed significant benefits or mixed results (Moniz-Cook et al., 1998; Onder et al., 2005; Clare et al., 2010; Prick et al., 2017). This echoes the review by Kurz et al. (2011) that sufficient duration is needed to support learning and implementation of strategies as learning abilities are affected in PLWDs. Carrion et al. (2018) further suggested that cognitive interventions potentially require a longer duration of more than 1 year to have effect.

The duration of follow-up measurement was observed to be a critical feature. Only one study observed a significant change of caregivers' mood among those in the control group at 6 months post-intervention relative to 3 months after intervention (Moniz-Cook et al., 1998) albeit the change was a deterioration in all measures of depression and anxiety. However, this result highlighted the need to measure caregivers' mood for a longer period before changes in the caregivers' mood can be seen post-intervention. The study had initially reported lower mood levels among the caregivers in the intervention group as compared to the control group at 3 months post-intervention. Given time, the trend was reversed at 6-month post-intervention: caregivers' mood in the intervention group remained fairly stable or slightly improved whereas the mood of caregivers in the control group had deteriorated (Moniz-Cook et al., 1998). To establish the role of time in the outcome of PLWD's caregivers warrants future research as such longitudinal studies are few (Ornstein and Gaugler, 2012).

### Focused intervention for behavioral disturbance

One observation in the current review was that studies with behavioral disturbance as primary outcome yielded benefits (Gitlin et al., 2008, 2018; Holden et al., 2019; O'Connor et al., 2019). Current principles of effective behavioral symptoms management emphasize the customization of interventions according to the understanding of the behaviors, triggers and contexts unique to each PLWD (Braun, 2019). Studies where behavioral disturbance is not the primary outcome may be less focus on understanding these elements. This can be seen in studies where behavioral disturbance was secondary outcome lacking personalized assessments to understand the needs and contexts of the PLWDs. Consequently, failure to understand dyadic needs hinders interventions from sufficiently targeting these issues, which Brodaty and Arasaratnam (2012) identified as a crucial component for success in behavioral symptoms interventions.

### Limitations in the outcome measures of PLWDs' mood

This review did not find any effect of home-based, non-exercise interventions on mood of PLWDs across all studies. This finding contrasts with the strong evidence for dyadic interventions in improving mood for community-dwelling PLWDs in another review (Van't Leven et al., 2013). One difference observed in current review is that the participants in all studies generally did not report high depression or anxiety scores at baseline. The absence of negative mood symptoms might limit the interventions from demonstrating effects in improving mood.

It was observed that none of the studies investigated positive mood and were mostly centered on depressive symptoms. Beerens et al. (2018) similarly highlighted this situation across literature on PLWDs' mood whereby negative mood is more frequently reported compared to positive mood, while Clarke et al. (2020) acknowledged that there is greater bias toward investigating mood disorders according to traditional deficit-centered paradigms. It is important to understand how interventions can promote positive mood as an essential aspect of wellbeing in PLWDs (Beerens et al., 2018) albeit without mood disorders. Furthermore, failure to use outcomes for positive mood may neglect to detect pleasure experienced by participants which was reported in Prick et al. (2016). This can potentially obscure the true effect of interventions on the mood of PLWDs.

### Difference in subjective and objective measures of caregivers' burden

It was observed that one study reported mixed results between objective and subjective caregivers' burden levels (Gitlin et al., 2008). As both measures are qualitatively different, it is unsurprising to find different results in the two caregivers' burden measures (Wolfs et al., 2012). It is noteworthy that the improvement in the objective caregivers' burden was measured in terms of time spent in caregiving, which can be reduced as the caregivers repeatedly practice carrying out the activities in the interventions and better adapt to other caregiving duties. The increased familiarity with caregiving tasks overtime may have then led to decreased time spent in caregiving objective but render no change to the subjective burden.

### Effectiveness of specific interventions on PLWDs' cognition

The current review did not find evidence for the effect of home-based ICST on cognitive outcomes, contrary to literature that cognitive stimulation therapy (CST) benefits cognition by delaying deterioration in cognitive reserves of PLWDs through intellectual and social stimulation (Duan et al., 2018; Chen et al., 2019). The discrepancy was attributed to a lack of social stimulation in individualized treatment and low intervention dose from poor adherence of caregivers in implementing ICST that resulted in lesser sessions per week (Orgeta et al., 2015; Orrell et al., 2017). However, Gibbor et al. (2021) reported significant cognitive improvements with a similar ICST programme conducted at a care home twice weekly by healthcare professionals. This suggests that ICST may potentially elicit cognitive gains in PLWDs but is influenced by the intervention dose rather than lack of social stimulation. Additionally, Cove et al. (2014) supported that once weekly CST is insufficient and recommended a frequency of twice weekly sessions for significant cognitive benefits. Hence, future studies could investigate the frequency of home-based ICST.

The current review also did not find evidence of home-based cognitive rehabilitation benefitting cognition as a secondary outcome despite improvement in functional abilities (Clare et al., 2010; Thivierge et al., 2014; Bourgeois et al., 2015). The greater brain activation patterns in face-name association tasks found with no significant improved cognition behaviorally (Clare et al., 2010) may likely be task-specific and not generalizable to overall cognition as cautioned by Roalf et al. (2014). This echoes the stance by Kurz et al. (2011) that benefits are specific to trained tasks and not generalizable to general cognition. Arguably, De Vreese et al. (2001) suggested that cognitive rehabilitation primarily aims to improve daily functional performance and not remediate cognitive abilities.

### Limitations of current review

All included studies were based in the Western context, hence the transferability of the findings to Asian contexts may be affected given the presence of different sociocultural factors in dementia care. The inclusion criterion of only using studies published in English could have further limited Asian studies published in different languages from being reviewed.

The studies had used varied outcome measures for specific outcomes. For example, caregiver burden was measured using various outcome measures such as the Zarit Burden Scale and the Caregiver Burden Inventory. Hence the lack of standardization might have affected the accuracy of analyzed results.

## Conclusion

There are mixed findings on the evidence of home-based, non-exercise interventions in alleviating PLWD's behavioral symptoms, functional status and cognitive decline as well as their caregivers' QoL. Evidence for improving the PLWDs' mood and their caregivers' burden and mood is limited.

Interventions that were tailored to the interest and abilities of the PLWDs alleviated their behavioral disturbance and functional decline. Longer interventions that incorporated reinforcement of strategies and involvement of caregivers in interventions benefits the PLWDs' cognition.

When interventions actively involved caregivers and included caregiver education and concurrently yielded a positive impact on the functional abilities of the PLWDs, benefits to the caregivers' QoL and burden were observed. However, the caregivers' involvement must not be overly intensive or causing inconvenience lest increasing their burden.

## Data availability statement

The original contributions presented in the study are included in the article/supplementary material, further inquiries can be directed to the corresponding authors.

## Author contributions

DT, BB, CC, and MT conceptualized the presented work. BB, CC, and MT conducted the systematic search, the title and abstract screening, the full text screening, extracted the data, conducted the reporting quality assessment, and drafted the first version of the manuscript as a report. DT supervised the project during each stage of the work. DT and B-SW drafted the manuscript for submission. All authors revised the manuscript for intellectual content and approved the final version of the manuscript.

## Conflict of interest

The authors declare that the research was conducted in the absence of any commercial or financial relationships that could be construed as a potential conflict of interest.

## Publisher's note

All claims expressed in this article are solely those of the authors and do not necessarily represent those of their affiliated organizations, or those of the publisher, the editors and the reviewers. Any product that may be evaluated in this article, or claim that may be made by its manufacturer, is not guaranteed or endorsed by the publisher.

## Footnotes

^1^Only unique participants were counted: two publications belonged to the same experiment of 273 dyad participants (Orgeta et al., 2015; Orrell et al., 2017) and another two publications belonged to the same experiment of 111 dyad participants (Prick et al., 2016, 2017). These publications reported different outcome measures; hence they were counted as distinct studies.
